# A Central Edge Selection Based Overlapping Community Detection Algorithm for the Detection of Overlapping Structures in Protein–Protein Interaction Networks

**DOI:** 10.3390/molecules23102633

**Published:** 2018-10-13

**Authors:** Fang Zhang, Anjun Ma, Zhao Wang, Qin Ma, Bingqiang Liu, Lan Huang, Yan Wang

**Affiliations:** 1 Key Laboratory of Symbol Computation and Knowledge Engineering of Ministry of Education, College of Computer Science and Technology, Jilin University, Changchun 130012, China; jlu_zhangfang@163.com (F.Z.); wzl3l452l@163.com (Z.W.); 2 Bioinformatics and Mathematical Biosciences Lab, Department of Agronomy, Horticulture and Plant Science, South Dakota State University, Brookings, SD 57007, USA; Anjun.Ma@sdstate.edu (A.M.); qin.ma@sdstate.edu (Q.M.); 3 Department of Mathematics and Statistics, South Dakota State University, Brookings, SD 57007, USA; 4 School of Mathematics, Shandong University, Jinan 250100, China; bingqiang@sdu.edu.cn

**Keywords:** protein–protein interaction network, overlapping community detection, central edge selection, overlapping node pruning

## Abstract

Overlapping structures of protein–protein interaction networks are very prevalent in different biological processes, which reflect the sharing mechanism to common functional components. The overlapping community detection (OCD) algorithm based on central node selection (CNS) is a traditional and acceptable algorithm for OCD in networks. The main content of CNS is the central node selection and the clustering procedure. However, the original CNS does not consider the influence among the nodes and the importance of the division of the edges in networks. In this paper, an OCD algorithm based on a central edge selection (CES) algorithm for detection of overlapping communities of protein–protein interaction (PPI) networks is proposed. Different from the traditional CNS algorithms for OCD, the proposed algorithm uses community magnetic interference (CMI) to obtain more reasonable central edges in the process of CES, and employs a new distance between the non-central edge and the set of the central edges to divide the non-central edge into the correct cluster during the clustering procedure. In addition, the proposed CES improves the strategy of overlapping nodes pruning (ONP) to make the division more precisely. The experimental results on three benchmark networks and three biological PPI networks of *Mus. musculus*, *Escherichia coli*, and *Cerevisiae* show that the CES algorithm performs well.

## 1. Introduction

The majority of the biological processes are constituted by a group of proteins which are connected densely [1]. The protein–protein interaction (PPI) network contains the communications among the protein groups that communicate with each other closely [2], which can be used to predict the complexity or function of normal proteins. The structures of the PPI networks can reflect some principles of the cellular organization [3]. Recently, the graph theory has been widely used to detect potential biological significance in PPI networks by regarding the proteins as nodes and the interactions between proteins as links [4,5,6]. Therefore, the procedure of finding new protein families can be converted to the procedure of detecting the sub-graph in PPI networks [7].

In the early 1930s, researchers began to detect community structure in sociology. Moreover, computational identification of special protein molecules is a key issue in understanding protein function [8]. To some extent, the community structure [9] can reflect the topological relations of real communities directly. The real communities, such as PPI networks in biology and World Wide Web (WWW) networks in sociology, tend to have the heavy-tailed power law, that is, only a small part of the nodes’ degrees is extremely higher than the rest. Therefore, network detection can be applied to the various research fields, such as biology and sociology [10,11,12,13,14].

Compared with non-overlapping communities, the overlapping community’s existence is more widespread in real communities, and comes up with many overlapping community detection (OCD) algorithms, such as CMC [15], MCL-Caw [16], and COACH [17]. In addition, many OCD algorithms which are based on the node calculation in regulatory social networks have had many achievements. In 2005, Palla et al. proposed the clique percolation method (CPM) based on the theory of mass infiltration to analyze the overlapping community structure of networks [18]. In 2011, Kim Y et al. proposed link clustering (LC) based on hierarchical clustering [19]. In 2014, Rodriguez et al. proposed a semi-supervised learning algorithm called density peaks clustering (DPC) to recognize cluster centers based on local density maxima [20]. Later in 2017, Qi Jinshan et al. proposed the OCD algorithm based on central node selection (CNS) to select the more reasonable central nodes [21]. Moreover, algorithms of OCD based on the edge information in regulatory social networks have also arisen. Evans et al. first utilized edge information to detect the overlapping network [22]. In 2010, Ahn Y-Y et al. proposed an OCD algorithm based on edge information to reveal the overlap and hierarchical structures in regulatory social networks [23]. Later in 2013, Lan Huang et al. proposed an extended link similarity (ELC), considering the similarity among the non-neighbor links [24]. Recently, an OCD algorithm was proposed based on links by combining a novel link similarity measure with the classic Markov cluster [25], and Deng X et al. proposed another algorithm combining the edge information with the Markov chain to detect overlapping communities [26]. The algorithms in clustering procedures and overlapping nodes pruning (ONP), such as the nearest neighbor algorithm (NN) [27] and community detection with an adjustable extent of overlapping [28], were developed dramatically. Beyond that, the evaluation algorithms for OCD, such as extended modularity (EQ) [29] and normalized mutual information (NMI) [30,31], have also arisen as needed.

In this paper, we introduce the CNS algorithm and propose an OCD algorithm based on central edge selection (CES), which could combine the advantages of edge selection with node selection. The algorithm consists of three major parts, the CES, the clustering procedure, and the overlapping nodes pruning. Firstly, the theory of community magnetic interference (CMI) was proposed in the process of CNS, considering the influence among the nodes. Then, we proposed the strategy of distance calculation between the non-central edge and the set of the central edge in the clustering procedure. Finally, we improved the algorithm of ONP to obtain more reasonable network divisions. The performance of the proposed algorithm was validated by comparing CES with CNS, CPM, and LC in three benchmark networks and three real PPI networks. The experimental results showed better evaluation values, such as EQ, NMI, and cover rate (CR), of CES in most situations according to the network division. More meaningful divisions can be achieved from CES than the original CNS, the traditional CPM algorithm, and traditional LC algorithm. In Section 2, the relevant algorithms including CNS, CES, the time complexity analysis of the CES, and evaluation methods are introduced. The experimental validation is presented in Section 3, and the conclusion is presented in Section 4.

## 2. Materials and Methods

### 2.1. Data Source

In order to assess the viability of CES and compare its performance with other algorithms, five real networks were selected, including three benchmark networks—Zachary’s Karate Club Network [32], Dolphins Social Network [33], and American College Football Network [9]—and two protein interaction networks—*E. coli* Network, *M. musculus* Network, and *Cerevisiae* Network (Table 1).

The first three benchmark networks describe community networks related to social communications or animal groups. (1) The *Karate network* dataset describes the interaction between every two members affected by two coaches in a karate club at a university in the United States. The nodes and edges refer to students and the communications among them, respectively. The resulting network includes 34 nodes and 78 edges. (2) The *Dolphin network* describes the relationship between two groups of bottlenose dolphins. After seven years of observation by Lusseau et al., a community including 158 edges and 62 nodes was obtained. Each edge represents the intersection between two dolphins, and the relationship in the community is relatively stable. According to the real situation, these dolphins can be divided into two categories. (3) The *Football network*, with 115 nodes and 612 edges, describes the rugby matches in 2000 between 12 different clubs and 115 teams. The nodes, edges, and categories represent different teams, the matches between every two teams, and the 12 clubs, respectively.

The other three datasets are as follows. (1) *E. coli*: This dataset describes the interaction between the proteins in *E. coli*. Each node in the network represents a protein, and an edge between the two nodes represents a relationship between the two proteins. The final network has 1396 nodes and 2092 edges. After removing these networks, the network with 344 nodes and 513 edges can be constructed. This dataset is a core protein interactive of the *E. coli* species, and the dataset name is Ecoli20170205. (2) *M. musculus*: This dataset describes the interaction between the proteins in *M. musculus*. Each node in the network represents a protein, and an edge between the two nodes represents a relationship between the two proteins. The final network has 1883 nodes and 2597 edges. After removing these networks, the network with 941 nodes and 1149 edges can be built. This dataset is a core protein interactive of the *M. musculus* species, and the dataset name is Mmusc20170205. (3) *Cerevisiae*: This dataset describes the interaction between the proteins in *Cerevisiae*. Each node in the network represents a protein, and an edge between the two nodes represents a relationship between the two proteins. The final network has 2172 nodes and 5124 edges. After removing these networks, the network with 2110 nodes and 4936 edges can be built. This dataset is a core protein interactive of the *Cerevisiae* species, and the dataset name is Scere20170205.

### 2.2. OCD Algorithm Based on Central Node Selection (CNS)

#### 2.2.1. Procedure of the CNS

In 2017, Qi Jinshan and Liang Xun proposed CNS to detect overlapping communities [21], which includes two main steps, the central node selection and the clustering procedure.

(1) In the first step, the exact central nodes can be achieved by evaluating the influence of a node. Suppose that a network G=(V,E) is given, where the V(G) and E(G) represent the set of nodes and edges in the graph G, respectively.

The definition of neighboring nodes of node *v* is set as the following formula:

(1) $$N(v)=\{v^{\prime }|(v^{\prime },v)\in E,\;v^{\prime }\in V\}$$

The definition $IB(v_{1},v_{2})$ of the influence between the node $v_{1}$ and the node $v_{2}$ is set as the following formula:

(2) $$IB(v_{1},v_{2})=\frac{D(v_{1})\times D(v_{2})}{d{\left(v_{1},v_{2}\right)}^{2}}=\frac{D(v_{1})\times D(v_{2})}{{\left(1-SIM(v_{1},v_{2})\right)}^{2}}$$

where D(v) represents the degree of node *v*, and $SIM(v_{1},v_{2})=\frac{N(v_{1})\cap N(v_{2})}{N(v_{1})\cup N(v_{2})}$ represents the Jaccard distance between node $v_{1}$ and node $v_{2}$.

The definition of all influence of node *v* is set as the following formula:

(3) $$ALL\left(v\right)=\sum_{\begin{array}{l} v_{n}\in N(v)\\ v_{n}\neq v \end{array}}IB(v,v_{n})=\sum_{\begin{array}{l} v_{n}\in N(v)\\ v_{n}\neq v \end{array}}\frac{D(v)\times D(v_{n})}{{\left(1-SIM(v,v_{n})\right)}^{2}}$$

The strategy of the central node selection is that if all influence of the node v is more significant than its neighbors, then it is selected as a central node in the community.

(2) In the second step, the non-central nodes can be clustered into the correct categories. Such a clustering procedure extends the communities, which are initialized from each central node. The relationship between a community and nodes is defined as the following formula:

(4) $$attract(EC_{i},u)=\frac{\sum_{v\in EC_{i}\wedge v\in N(u)}IB(u,v)}{ALL(u)}$$

where $EC_{i}$ represents a community needing to be extended, *u* represents the neighbor nodes of $EC_{i}$, and *v* represents both the neighboring nodes of *u* and the nodes in $EC_{i}$. The neighboring nodes of $EC_{i}$ can be enriched by adding nodes with an *attract* value higher than the threshold ε=0.4 [21]. As a result, the new community can be achieved by iterating the search-and-add of neighboring nodes.

#### 2.2.2. Limitation of CNS

**Figure 1:**
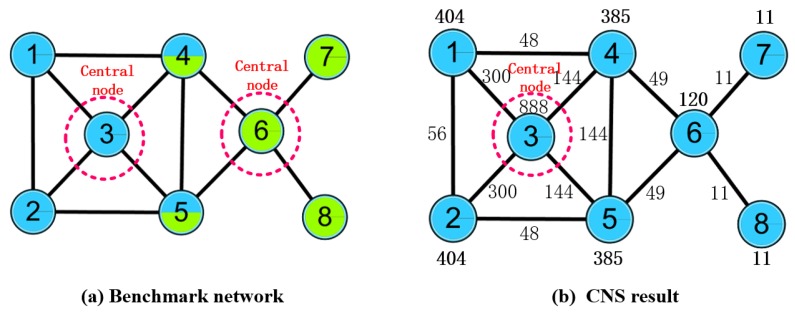
Example of the limitation of the central node selection (CNS) algorithm.

Although the OCD algorithms based on central node selection have many advantages in detecting overlapping communities, such as combining the local information and global information of the regulatory social networks, the accuracy of the central node selection and the overlapping degree of the networks still hold the potential to be expanded. Specifically, considering the fact that the process of central node selection only focuses on the node itself and ignores the influence among the nodes, it may lead to CNS being incorrect. Many constraints should be considered for the formation of the overlapping nodes in each community they belong to, which leads to difficulty in using CNS to achieve overlapping nodes. Therefore, the degree of the overlapping node is insufficient in CNS. In either case, the result of the community detection can hardly match the real network well. For instance, in a small benchmark demo network containing 8 nodes and 12 edges (Figure 1a), the ALL(v) values (Figure 1b) can be calculated by the CNS algorithm, and node 3 will be regarded as the only central node. While in the benchmark network, two central nodes, node 3 and node 6, will be considered as central nodes.

### 2.3. OCD Algorithm Based on Central Edge Selection (CES)

**Figure 2:**
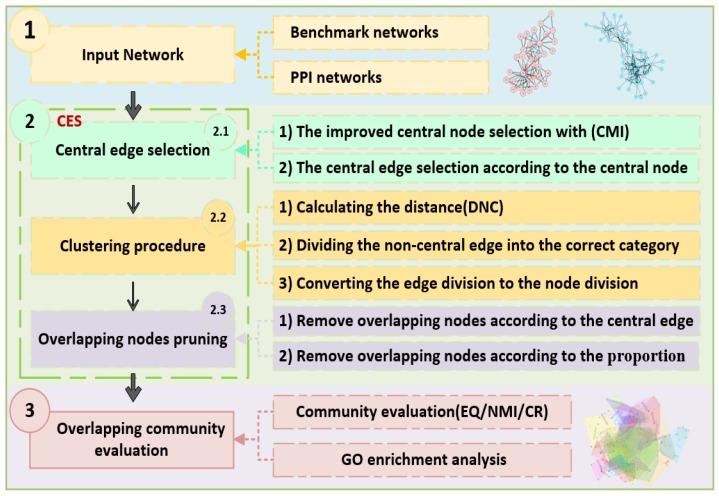
Workflow of the CES algorithm.

To avoid the shortcomings of CNS, we proposed CES using the information of edges to detect the overlapping communities. The workflow of the CES algorithm shown in Figure 2 contains three major parts, including a procedure of central edge selection, a clustering procedure, and an ONP step. The theory of CMI, introduced in Section 2.3.1, takes into consideration the influence among nodes to make the target central node more reliable. The network can be divided by edges to reduce the difficulty of getting overlapping nodes, and then optimized by ONP.

#### 2.3.1. Central Edge Selection

The process of central edge selection is composed of two parts: An improved central node selection integrated with CMI, and the central edge selection.

(1) In the first part, the CMI theory is used to improve the process of the central node selection, which alters the central nodes to affect their neighboring nodes. Here, a formula used to revise the *ALL* value of nodes is shown as follows:

(5) $$ALL\left(v\right)=GF\times \sum_{u\in N(v)}IB(v,u)$$

where *v* and *u* refer to the confirmed central node and its neighboring nodes, respectively. GF is a coefficient used to revise the *ALL* value of nodes according to CMI.

The influence between nodes in the network is calculated using Formula (2), and updates the *ALL* value by Formula (5), after determining one central node using the strategy of CNS in the CNS algorithm.

The pseudo-code of the improved central node selection can be described as following Algorithm 1:
**Algorithm 1.** Improved central node selection.1 *Calculate the all influence of nodes*2 For each v∈V do3  $ALL\left(v\right)=\sum_{\begin{array}{l} v_{n}\in N(v)\\ v_{n}\neq v \end{array}}IB(v,v_{n})=\sum_{\begin{array}{l} v_{n}\in N(v)\\ v_{n}\neq v \end{array}}\frac{D(v)\times D(v_{n})}{{\left(1-SIM(v,v_{n})\right)}^{2}}$
4 End for 5 *Central node selection with CMI*6 For each v∈V do7  *Central node selection*8  If $\forall v^{\prime }\in N(v)\&\forall v^{\prime }\notin N(CN)$ and $ALL\left(v^{\prime }\right)\leq ALL\left(v\right)$
9  Then10   CN=CN∪{v}
11   ALL(v)=0
12   *Revise according to the CMI*13   For each $v_{v}\in N(v)$ do14    $ALL\left(v_{v}\right)=GF\times \sum_{u\in N(v_{v})}IB(v_{v},u)$15   End for16 End for
where *CN* refers to the set of central nodes. The N(CN) represent all the neighboring nodes of the confirmed central nodes, which reduces the possibility of two adjacent nodes becoming the central nodes together; as a result, the case where two adjacent nodes are central nodes together cannot occur in the real network.

(2) In the second part, after selecting the central nodes, the procedure of the central edge selection is to classify all the edges connected with the central node as the central edges, and the remaining edges are classified as the non-central edges.

For each central node, the central edges category (CEC) is determined by $CEC(CE_{i})=i$, where $CE_{i}=\{e(v_{1},v_{2})|v_{1}=v\;or\;v_{2}=v\}$ represents the set of the Central Edges linked to a central node with *i* index, and $e(v_{1},v_{2})$ represents the edge between node $v_{1}$ and node $v_{2}$. Edges other than the central edges are classified as non-central edges.

**Figure 3:**
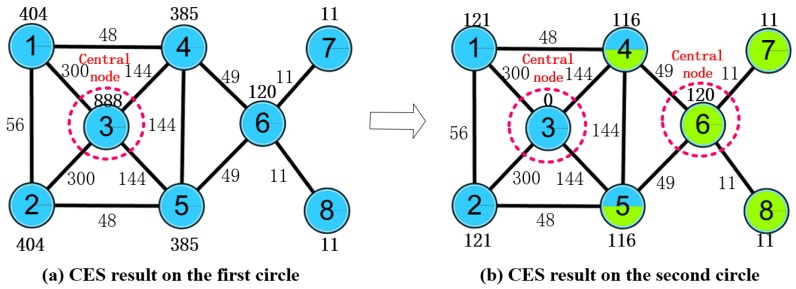
An example of a CES result.

Considering the same demo network constructed in Section 2.2.2, more reasonable results can fortunately be achieved after recalculating the benchmark network (Figure 1a) with the CES algorithm. In the first circle, we calculate the ALL(v) value (Figure 3a), which is the same as the CNS results (Figure 1b), and regard node 3 as the first central node. Then the values of node 3’s neighboring nodes are revised (Figure 3b) according to the theory of CMI, which is introduced in the following Section 2.3.1. Hence, the other central node, node 6, can be selected, as a result of which the values of node 6’s neighboring nodes are smaller than node 6, and node1, node 2, node 4, and node 5 are not taken into account. Then, two overlapping nodes can be selected—node 4 and node 5. The result is the same as the benchmark network division (Figure 1a).

#### 2.3.2. Clustering Procedure

The clustering procedure intakes the result from the procedure of central edge selection to categorize the non-central edges by three steps: Calculating the distance between the non-central edge and the central edges, allocating the non-central edge into the correct category, and converting the edge division into the node division.

(1) In the first part, a novel edge similarity measure $ELC(e_{k},e_{j})$ [24] is defined as follows to calculate the distance between the edges with edge information.

(6) $$ELC(e_{k},e_{j})=ELC(e(a,b),e(c,d))=\left|\frac{N(a)\cap N(c)+N(a)\cap N(d)+N(b)\cap N(c)+N(b)\cap N(d)}{N(a)\cup N(c)+N(a)\cup N(d)+N(b)\cup N(c)+N(b)\cup N(d)}\right|$$

where e(a,b) represents the edge e(a,b) which has two nodes, node *a* and node *b*; and N(a) represents the neighboring nodes of node *a.* Therefore, the distance between the non-central edge $e_{k}$ and the set of the central edges in $CE_{i}$ can be defined as $DNC(e_{k},CE_{i})$:

(7) $$DNC(e_{k},CE_{i})=\sum_{e_{j}\in CE_{i}}\frac{ELC(e_{k},e_{j})\times (\sum_{e_{m}\in CE_{i}}ELC(e_{k},e_{m})-ELC(e_{k},e_{j}))}{\sum_{e_{m}\in CE_{i}}ELC(e_{k},e_{m})}$$

where the $e_{m}$ and $e_{j}$ represent the central edges belonging to the categories *i*.

(2) After calculating all the distances $DNC(e_{k},CE_{i})$ of $e_{k}$, the minimum value of $DNC(e_{k},CE_{i})$ can be found, and the non-central edge $e_{k}$ belongs to the corresponding category *i* based on the NN algorithm [27].

(3) Finally, the remaining edge divisions are converted to the node division. The category of each edge and the corresponding two nodes in the network are the same. In this way, the node division of the network can be achieved as the final result.

#### 2.3.3. ONP Procedure

In this paper, we have improved the ONP algorithm [28] by mixing two strategies. The two strategies are related to each other, and the first strategy is the special case of the second strategy, which can eliminate some steps of pruning and save running time of the CES algorithm.

(1) In the first strategy, overlapping nodes, whose connections are central edges completely in some categories, can be removed from some categories; that is, $con(v_{i},C_{j})\in CE_{i}$, where $C_{j}$ represents the edges in the category *j*, and $con(v_{i},C_{j})$ represents the connections between the central node $v_{i}$ and $C_{j}$. It is not necessary to calculate the number of non-central edges between central nodes and categories.

**Figure 4:**
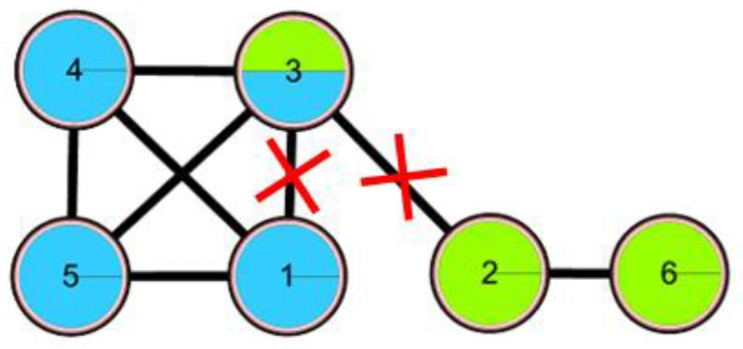
An example of the first pruning strategy.

For the example in Figure 4, suggest that node 1 and node 2 are central nodes and node 3 is the overlapping node. According to the first strategy, node 3 can be changed to the left category only; that is, the connection between node 3 and the right category is the edge 2 to 3, which is completely the central edge.

(2) In the second strategy, the connections of each overlapping node in different categories have a different proportion, and overlapping nodes whose proportion is less than prop can be removed; that is, $\frac{con(v_{i},C_{j})}{\sum_{k\in clus(v_{i})}con(v_{i},C_{k})}<prop$, where $clus(v_{i})$ represents the categories of the node $v_{i}$, and the empirical value prop represents the threshold during the ONP.

**Figure 5:**
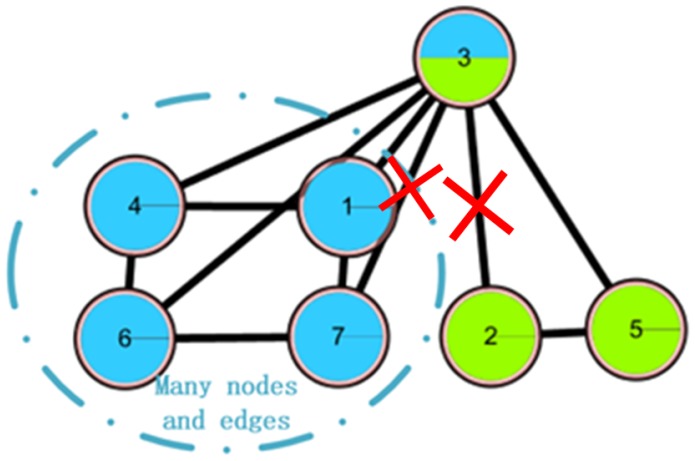
An example of the second pruning strategy.

A simple network is shown in Figure 5 in which node 2 and node 7 are central nodes and node 3 is the overlapping node. The connection between node 3 and the right category has only one non-central edge, while the connection between node 3 and the left category has many non-central edges. Therefore, node 3 will be included in the left category only.

#### 2.3.4. Time Complexity Analysis

If the network is scale-free, such as the PPI network, then the network obeys the power-law distribution [34]. Suppose *n* represents the number of nodes, *m* represents the number of edges, the *seed* represents the number of central nodes, and adj(i) represents the number of node *i*’s neighboring nodes. In the procedure of the central edge selection, time is mainly spent in calculating the *all* values of all nodes, which is $O(n^{2})$ based on Formula (3), improving central nodes selection based on CMI, which is O(n×adj(i)) according to the improved CNS pseudo-code, and the selection of central edges based on the central node, which is O(n). In the clustering procedure, time is mainly spent in dividing the non-central edges into appropriate categories, which is $O(seed\times m^{2})$ according to Formulas (6) and (7). In the ONP procedure, time is mainly spent in finding connections of overlapping nodes in different categories, which is O(n×m). In the power-law distribution, the degree of each node is the probability of a natural number *k* where $P(\deg ree=k)\propto \frac{1}{k^{\gamma }}$; that is, if a node’s degree is *k*, then the probability is $\frac{1}{k^{\gamma }}$. In 2001, Béla Bollobás et al. found the γ=3 in a big network [35]. The degree of the network is $DN=1\times \frac{1}{1^{3}}+2\times \frac{1}{2^{3}}+\ldots +n\times \frac{1}{n^{3}}\leq \frac{6}{\pi^{2}}\times n$, and the number of the edges is $m=\frac{DN}{2}\leq \frac{3}{\pi^{2}}\times n$. So, the final time complexity is $O(n^{2}+seed\times m^{2}+n+n\times adj(i)+n\times m)$, that is $O(n^{2})$. From Table 2, the comparison of the algorithms’ running times can be seen clearly.

### 2.4. CPM Algorithm

In 2005, Palla et al. proposed CPM based on the theory of mass infiltration to analyze the overlapping community structure of networks [18]. The result of CPM is based on the conception of K-cliques, which represents the K nodes connected with each other, and the two K-cliques are adjacent if they have (K − 1) common nodes. If K is given, CPM can search all adjacent K-cliques in the networks starting from any K-cliques, and these adjacent K-cliques are divided into the same cluster. Then CPM starts from any K-cliques which are not divided, and starts iteration by searching all adjacent K-cliques. The CFinders package [18] (version 2.0.6, Eötvös University, Budapest, Hungary) is supposed to get the process of the CPM.

### 2.5. LC Algorithm

In 2011, Kim Y et al. proposed LC based on hierarchical clustering [19]. The advantage of LC is that the node community scheme and link community scheme can be compared quantitatively by measuring the unknown information left in the networks besides the community structure. It can be used to determine quantitatively whether link community schemes should be used rather than node community schemes. However, LC easily achieves the local minimum and tends to divide the communities into small clusters.

### 2.6. Evaluation

To evaluate the performance of our CES based algorithm we used three evaluation standards, EQ, NMI, and CR, to compare with the performance of CNS and CPM. Specifically, for the PPI network, an additional Gene ontology (GO) enrichment analysis was introduced to evaluate the biological meaning of the network constructed by the four algorithms.

#### 2.6.1. EQ Algorithm

In 2004, Newman et al. proposed an evaluation algorithm module Q, which can be used to evaluate the result of non-overlapping community detections, though it is not suitable to detect overlapping communities. In order to amend the algorithm, in 2009, Shen et al. proposed a novel evaluation EQ algorithm [29].

For a given network G=(V,E), the community $C=\{C_{1},C_{2},\ldots ,C_{i}\}\;i=1,2,\ldots ,m$ contains *m* categories, and EQ can be defined as below.

(8) $$EQ=\frac{1}{2\times link\_num}\sum_{i}\sum_{v\in C_{i},w\in C_{i}}\frac{1}{CN_{v}CN_{w}}\left(EB_{vw}-\frac{D(v)\times D(w)}{2\times link\_num}\right)$$

In the formula, *m* refers to the number of edges in each community, and $CN_{v}$ and $CN_{w}$ refer to the number of categories that node *v* and node *w* belong to, respectively. $EB_{vw}$ is a logical value that represents the existence status of the edge between node *v* and node *w*; 1 for existent and 0 for missing. D(v) and D(w) represent the degree of node *v* and node *w*, respectively. The EQ value ranges from 0 to 1, and a higher value indicates closer structure to the standard division. In the exceptional case, when the result of the community structure is identical to the original standard division, the EQ value is 1.

#### 2.6.2. NMI Algorithm

In 2009, Lancichinetti et al. proposed a novel evaluation algorithm called NMI [30,31], which evaluates the accuracy between the CES result and the standard division. The NMI score ranges from 0 as completely different, to 1 as identical. The following equation defines NMI:

(9) $$NMI(X|Y)=1-\frac{1}{2}[H{\left(X|Y\right)}_{norm}+H{\left(Y|X\right)}_{norm}]$$

where *X* refers to the standard division of the community and *Y* refers to the CES constructed community division. $H{\left(X|Y\right)}_{norm}$ and $H{\left(Y|X\right)}_{norm}$ are the normalized condition entropy of *X* with respect to *Y*, with $H{\left(X|Y\right)}_{norm}=\frac{1}{\left|NC\right|}\sum_{k}\frac{H(X_{k}|Y)}{H(X_{k})}$, where *NC* represents the number of categories in the network, and $X_{k}$ represents the network of category *k*; and $H{\left(Y|X\right)}_{norm}$ is likewise.

#### 2.6.3. CR Algorithm

The CR is used to describe the coverage of nodes in the community compared to those in the original community. It can be defined as $CR=100\times \frac{n^{\prime }}{n}$, where $n^{\prime }$ refers to the number of nodes in the produced community division, and n refers to the number of nodes in the original.

#### 2.6.4. GO Enrichment Analysis

In biological network study, GO is a common method used to compare the proteins (or genes) in a predicted network to the known universal functional groups with annotations, and evaluates how close the connections are. Three major aspects are involved in the GO analysis: (1) Biological process (BP) compares the functions or final outcomes of proteins from specific gene sets that carry the same function; (2) molecular function (MF) describes the biochemical activity of the given protein’s sets; and (3) cellular component (CC) emphasizes the relative proteins location in a cell and cellular anatomy. For each of the GO enrichment analyses, the *p*-value is calculated to evaluate the probability predicted protein modules match the protein list annotated to the particular terms. Significant *p*-values indicate strong association of the proteins with a group. In this paper, we adopt the *p*-value provided by the R-package ClusterProfiler [36] to analyze the PPI network division.

## 3. Results and Discussion

### 3.1. Benchmark Network

The four OCD algorithms (CES, CNS, CPM, and LC) were tested using the three benchmark networks (*Karate*, *Dolphin,* and *Football*), and computational networks were evaluated by three criteria (EQ, NMI, and CR). The evaluation results of four OCD algorithms on three benchmark networks can been seen from Table 3.

**Figure 6:**
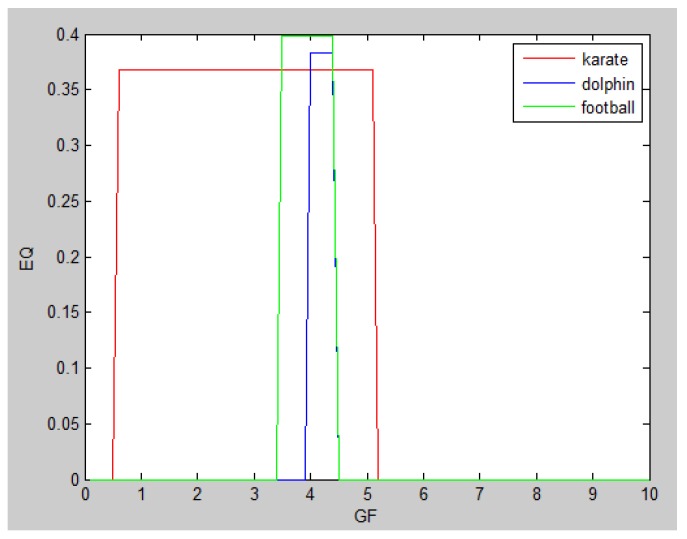
The selection of *GF*.

During the procedure of central edge selection, GF=4.2× node_num_/edge_num_ and prop= node_num_/edge_num_ during the overlapping nodes pruning, where *node_num* represents the number of nodes in the network and *edge_num* represents the number of edges in the network. Figure 6 represents the selection of *GF*, which is based on the value of EQ on the three networks. *GF* is finally selected as 4.2 × node_num_/edge_num_. BCN refers to the number of categories on benchmark networks that are recorded in each publication, and evaluation category number (ECN) represents the number of the category which is produced from the algorithms. The bold numbers are the best values among all algorithms.

For the three datasets, the CES method achieved high scores for all three evaluations, and most of them surpassed the CNS, CPM, and LC methods. Furthermore, the ECN described by CES were identical to the known BCN.

In the *Karate Network*, CES has a better result than CNS, CPM, and LC for all three evaluation methods. The EQ value is 0.37 and the NMI value is 0.92. In addition, CES has a total cover rate. Additionally, the division of the CES has two categories, which is the same as the standard category. In the *Dolphin Network*, CES has a better result than CNS, CPM, and LC in NMI, with a value of 0.76. CES’s EQ value is 0.38, which is slightly lower than CNS, as a result of which the number of the category CES has is the same as the number of the standard category, while CNS is inconsistent. Therefore, CNS is inaccurate in getting the correct number of categories, and the high EQ value of CNS has no significance. In addition, CES has a total cover rate. In the *Football Network*, CES has a better result than CNS, CPM, and LC in EQ, with a value of 0.4. CES’s NMI value is 0.52 which is slightly lower than CNS, as a result of which the number of the category CES has is the same as the number of the standard category, while CNS is inconsistent. Hence, CNS is inaccurate on getting the correct number of categories, and the high NMI value of CNS has no significance. In addition, CES has a 99% cover rate and is almost completely covered. The visualization of the four algorithms’ (CES, CNS, CPM, and LC) results on the three benchmark networks (*Karate Network, Dolphin Network*, and *Football Network*) is shown in Table 4, and Cytoscape [37] is used to visualize the network division. In addition, the results of LC on the three benchmark networks have big differences in the number of categories from the benchmark, so the results are meaningless and we do not show the results of LC.

### 3.2. PPI Network

**Figure 7:**
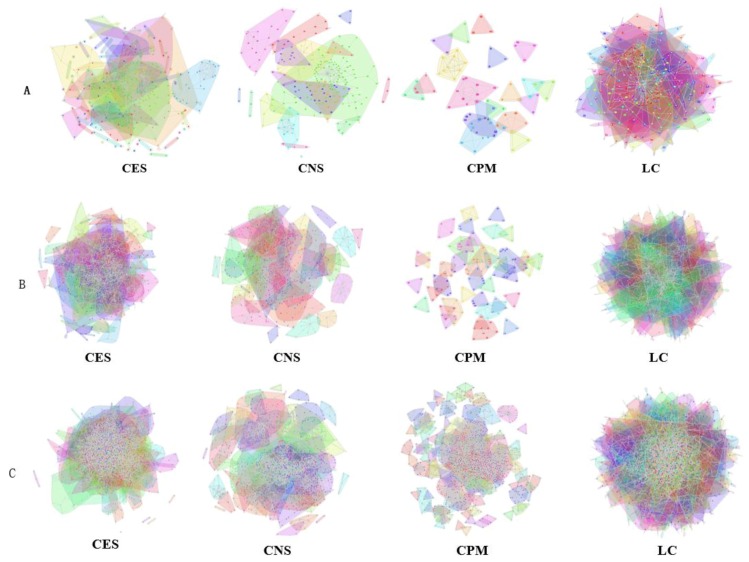
Visualization of the predicted PPI network using four algorithms. (**A**) *M. musculus* dataset. (**B**) *E. coli* dataset. (**C**) *Cerevisiae* dataset.

Three PPI networks, from *M. musculus*, *E. coli*, and *Cerevisiae,* were used to test and compare the performance of the four OCD algorithms (CES, CNS, CPM, and LC). The *GF* values used for each dataset were chosen as 0.9, 0.8, and 0.5, respectively, and 0.1 *prop* for among the datasets. In each dataset, the CES method showed higher EQ and CR than CNS, CPM, and LC (Table 5). The categories found by CES in the three datasets covered all nodes (proteins) in the population, while CNS only covered 65%, 72%, and 55%, respectively, LC only covered 78%, 60%, and 92%, respectively, and the CPM covered less. Table 5 displays all categories found by the four algorithms. The LC results show much more overlap among categories in each dataset, which induced higher network redundancy, and thus, is far away from the actual protein network structures. The visualization of the predicted PPI network using four algorithms can been seen from Figure 7.

By performing GO enrichment analysis, the *p*-values of BP, MF, and CC were calculated to evaluate the connections between the predicted categories and biological functional protein groups (see details in Supplementary Table S1). Considering the overall performance among algorithms, we considered categories (protein modules) with a *p*-value < 0.001 as significant, and the total number of significant categories are summarized in Table 6. For most cases, the number of significant categories predicted by CES was more than those from CNS, CPM, and LC; the CPM showed a higher rate of significant categories while only presenting a relatively local relationship due to the low CR results, and the LC algorithm excessively categorized the nodes that lead to higher numbers of the total and significant categories with higher biases. Nevertheless, combining with the overall CR, the CES algorithm still showed the best results for community categories prediction. The individual *p*-values were log-normalized and are distributed in Supplementary Figures S1–S3 in order to showcase the overall comparison among algorithms and datasets.

**Figure 8:**
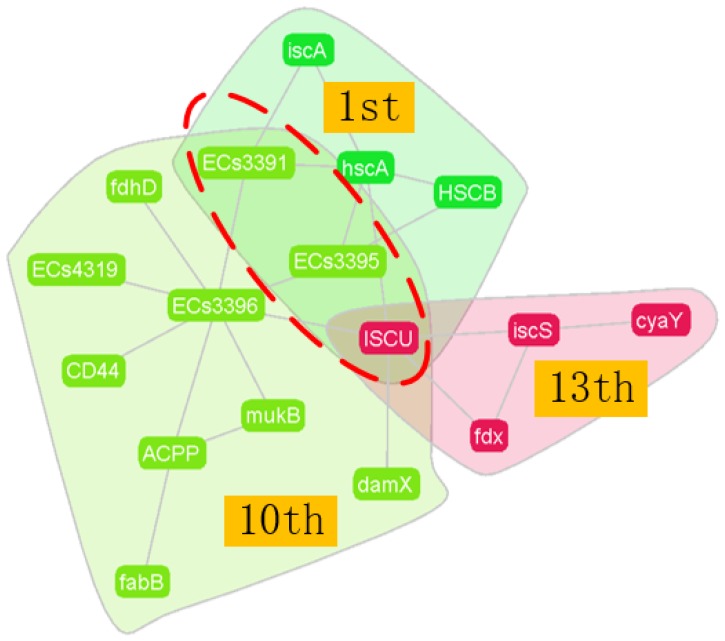
Overlapping structure of the No.1 category in *E. coli*.

Two categories predicted by the CES algorithm, No. 3 in *M. musculus* and No. 1 in the *E. coli* dataset, were selected to showcase the investigation of the relationships among categories and overlapped nodes. For the No.1 significant category in *E. coli*, six proteins, iscA, ECs3391, ECs3395, HSCB, hscA, and ISCU, were included. Protein hscA, responsible for the transfer of iron-sulfur clusters, was considered as the central node and contributed to the enriched category function. The No. 1 category was found to overlap with the 10th and 13th categories, and shared a common overlapping protein, ISCU, which assembles the Fe-S clusters. The 1st and 10th categories overlapped at two more protein positions, ECs3391 and ECs3395, other than ISCU. ECs3391 is an iron-sulfur protein that helps the assembly of Fe-S clusters, and ECs3395 is a scaffold protein that works with ISCU in the formation of Fe-S clusters. The overall relationships of the three categories are shown in Figure 8. The individual protein functions can be found in Supplementary Table S2, along with the overlapping investigation in *M. musculus*.

## 4. Conclusions

In this study, a CES based OCD algorithm was introduced to construct community networks. The improved CES method applies the CMI algorithm in the traditional central node selection step, and combines with central edge selection to use both nodes and edge information for the main community construction. Then, the clustering procedure calculates the distance between the non-central edge and central edge to allocate the non-central edges into the right categories. Finally, an improved ONP algorithm is applied to assign the overlapping nodes into an appropriate community to complete the network construction. To evaluate the performance of network construction, the proposed CES method was used to test three benchmark networks and two protein–protein interaction networks, and compared with the CNS, CPM, and LC methods. The results indicated excellent performance of the CES algorithm in the community with moderate complexities. As a result, we believe our CES algorithm has the potential to achieve more accurate and sufficient networks for community studies, especially in sociology and the systematic biology area. Our future work will focus on improving the efficiency and accuracy of the CES algorithm, and adapting it to dynamic network analyses.

## Figures and Tables

**Table 1:** Six real networks.

Dataset	Nodes	Edges	BCN
*Karate*	34	78	2
*Dolphin*	62	158	2
*Football*	115	612	12
*E. coli*	1396	2092	-
*M. musculus*	1883	2597	-
*Cerevisiae*	2172	5124	-

Benchmark networks categories number (BCN) refers to the number of categories on benchmark networks that are recorded in each publication.

**Table 2:** The comparison of the algorithms’ running times.

		Methods	*Karate*	*Dolphin*	*Football*	*E. coli*	*M. musculus*	*Cerevisiae*
	RT(s)							
Datasets								
CES			0.02	**0.105**	**0.413**	1.742	58.746	863.617
CNS			0.124	0.609	2.809	67.487	1395.1	15,780
CPM			**0.01**	0.3	0.8	**1**	**5**	**7**
LC			0.636	1.841	7.331	20.988	187	1682.22

The runtime in seconds (RT(s)) in the table represent the runtime and the bold numbers represent the best RT among all algorithms.

**Table 3:** The evaluation results of four overlapping community detection (OCD) algorithms (CES, CNS, clique percolation method (CPM), and link clustering (LC)) on three benchmark networks (*Karate Network*, *Dolphin Network,* and *Football Network*).

Dataset	*Karate*					*Dolphin*					*Football*				
Evaluation	EQ	NMI	CR	BCN	ECN	EQ	NMI	CR	BCN	ECN	EQ	NMI	CR	BCN	ECN
CES	0.37	0.92	100%	2	2	0.38	0.76	100%	2	2	0.40	0.52	99%	12	12
CNS	0.35	0.69	100%	2	2	0.46	0.41	100%	2	3	0.28	0.62	44%	12	5
CPM	0.19	0.18	94%	2	3	0.36	0.32	74%	2	4	0.19	0.26	100%	12	4
LC	0.17	0.06	97%	2	12	0.18	1 × 10^−16^	87%	2	22	0.16	5.5 × 10^−17^	100%	2	46

**Table 4:** The visualization of three algorithms’ (CES, CNS, and CPM) results on three benchmark networks (*Karate Network, Dolphin Network*, and *Football Network*).

	Algorithms	CES	CNS	CPM
Datasets				
*Karate Network*		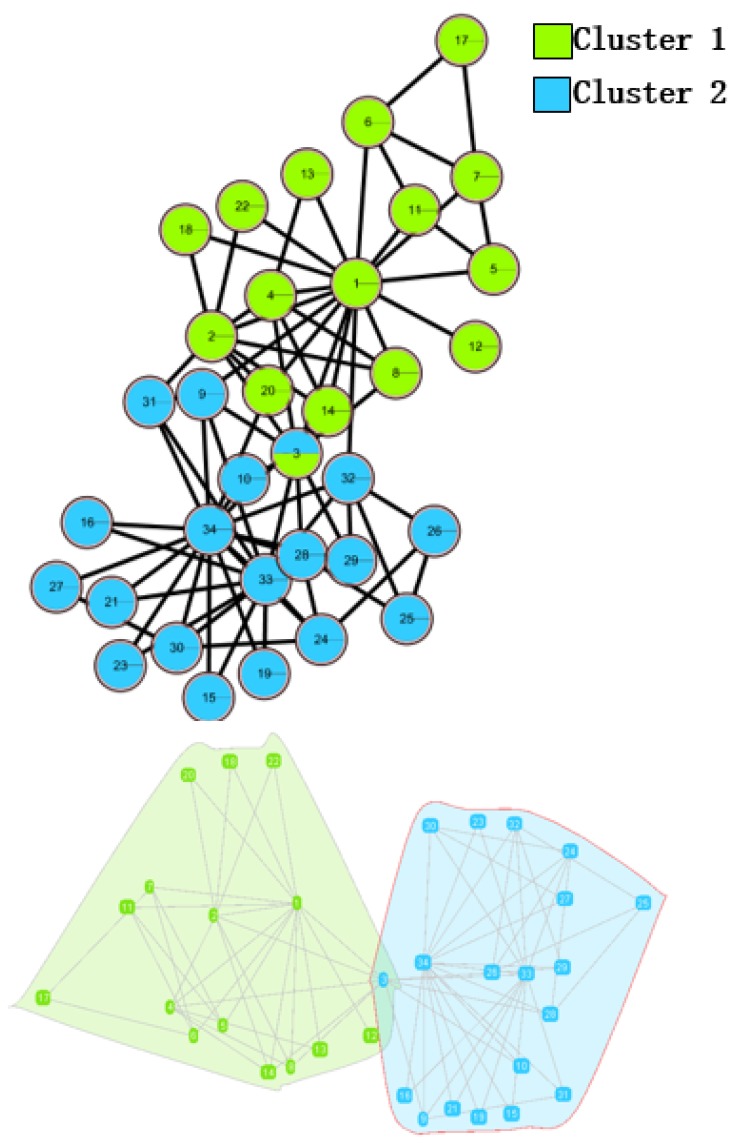	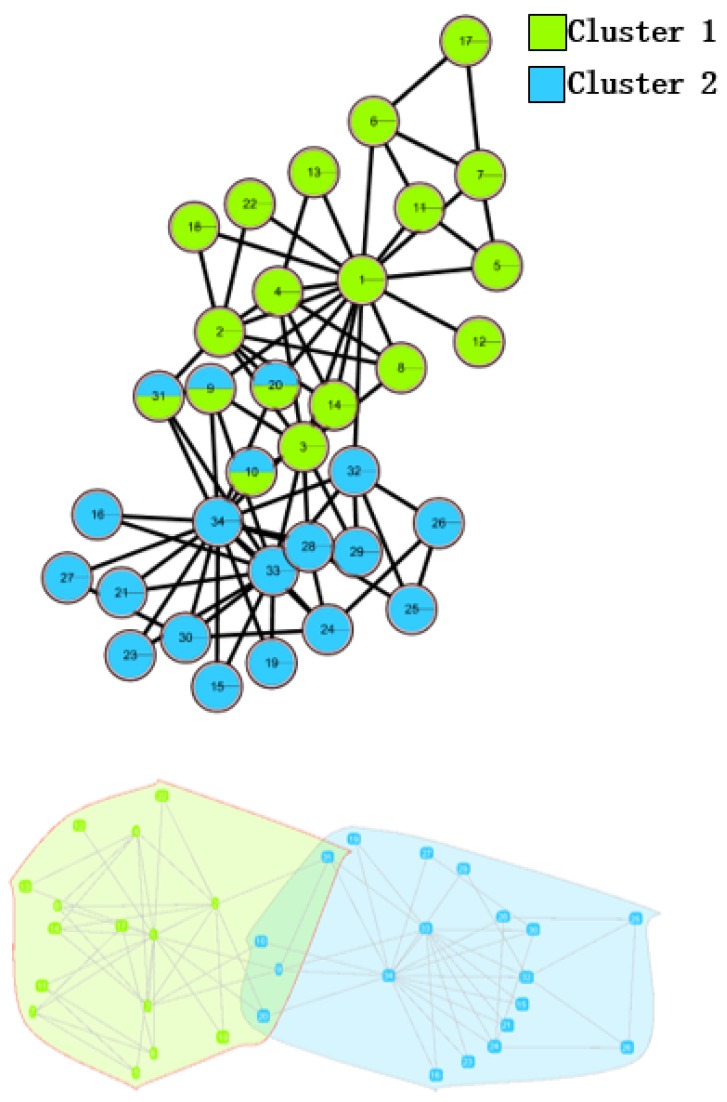	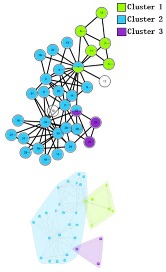
*Dolphin Network*		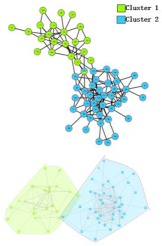	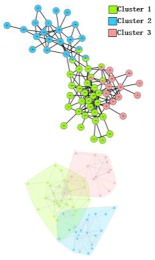	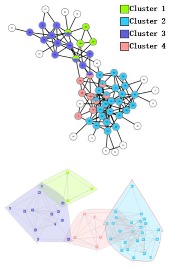
*Football Network*		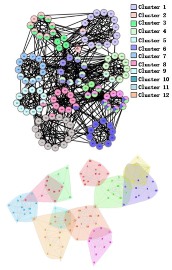	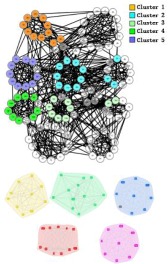	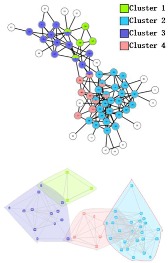

**Table 5:** The results of four algorithms (CES, CNS, CPM, and LC) on three Protein–Protein Interaction (PPI) networks (*M. musculus Network*, *E. coli Network*, and *Cerevisiae*).

Dataset	*M. musculus*			*E. coli*			*Cerevisiae*		
Evaluation	EQ	CR	ECN	EQ	CR	ECN	EQ	CR	ECN
CES	**0.719**	**100%**	85	**0.519**	**100%**	77	**0.562**	**100%**	105
CNS	0.534	65%	43	0.49	72%	18	0.438	55%	46
CPM	0.191	18%	41	0.226	23%	19	0.467	53%	161
LC	0.19	78%	149	0.10	60%	47	0.06	92%	580

The bold numbers represent the best result among all algorithms.

**Table 6:** Total number of significant categories with *p*-value ≤ 0.001 predicted by each algorithm.

Datasets	CES	CNS	CPM	LC
*M. musculus*	66/85	33/43	40/41	118/149
*E. coli*	44/77	17/18	15/19	10/47
*Cerevisiae*	79/105	44/46	159/161	344/580

## Supplementary Materials

Figure S1: Comparison of three levels on *M. musculus* Network, Figure S2: Comparison of three levels on *E. coli* Network, Figure S3: Comparison of three levels on *Cerevisiae* Network.
