# Supplementary material for: A Central Edge Selection Based Overlapping Community Detection Algorithm for the Detection of Overlapping Structures in Protein–Protein Interaction Networks
Source: Molecules. 2018 Oct 13;23(10):2633. doi: 10.3390/molecules23102633 (PMC6222769; doi:10.3390/molecules23102633)
Supplement: Supplementary file 1 [file molecules-23-02633-s001.zip › supplementary figure s1-s3.pdf]

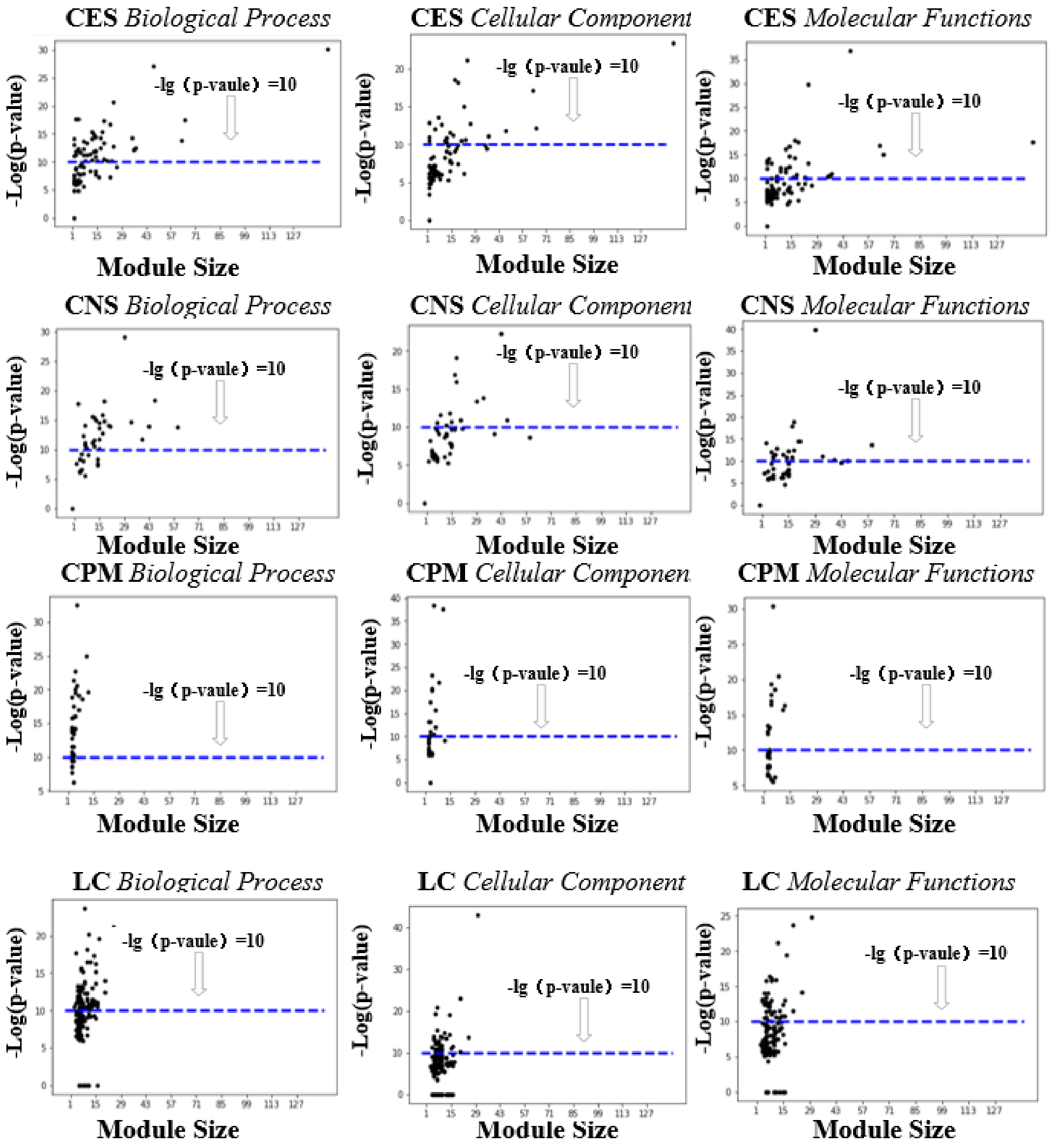

Figure S1 Comparison of three levels on *M. musculus Network*

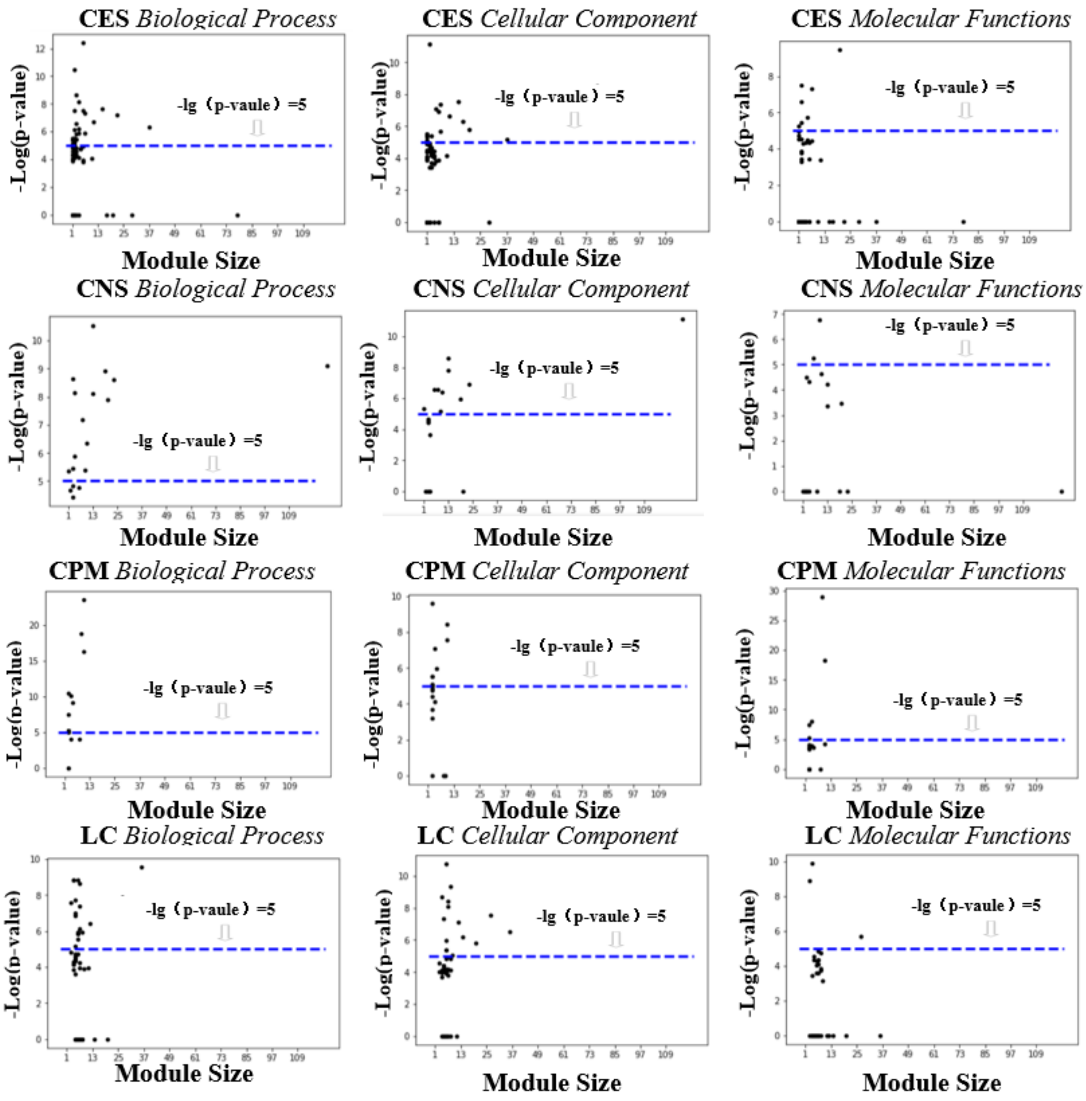

Figure S2 Comparison of three levels on *E. coli Network*

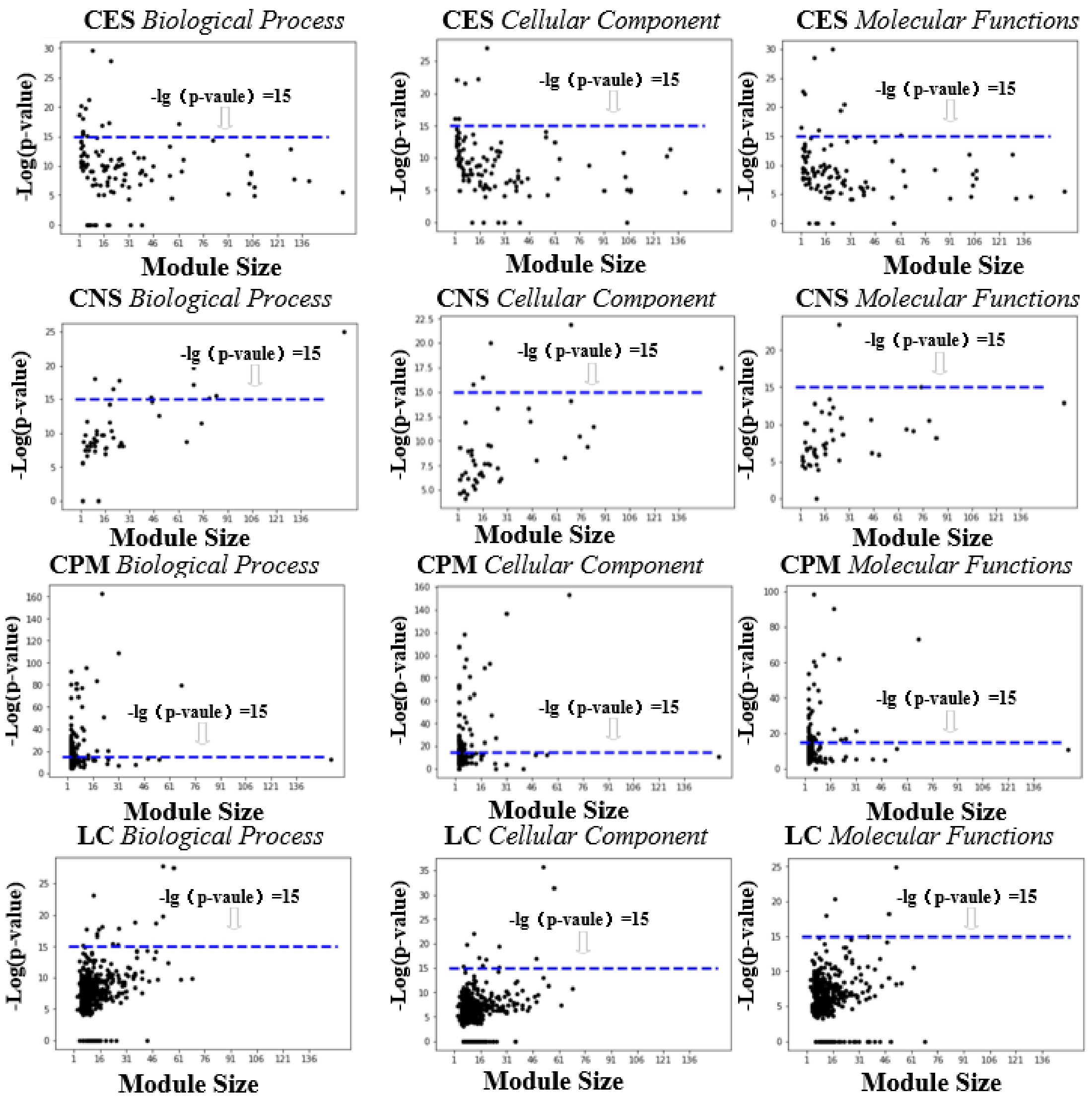

Figure S3 Comparison of three levels on *Cerevisiae Network*
